
JOURNAL INFORMATION
==============================
NLM Title Abbreviation: J Can Health Libr Assoc
Journal ID: J Can Health Libr Assoc
NLM Journal ID: 101229936
Journal ID: JCHLA

The Journal of the Canadian Health Libraries Association

EISSN: 1708-6892
Publisher: Canadian Health Libraries Association / Association des bibliotèques de la santé du Canada

ARTICLE INFORMATION
==============================
PMCID: PMC11485163
DOI: 10.29173/jchla29789
Article ID: JCHLA-45-123
Article version: 1
Subjects: Chla 2024 Conference Lightning Talks / Absc Congrès 2024 Présentations Éclair

CHLA 2024 CONFERENCE LIGHTNING TALKS / ABSC CONGRÈS 2024 PRÉSENTATIONS ÉCLAIR

Electronic publication date: 2024 Aug 1
Publication date: 2024 Aug
Volume: 45
Issue: 2
First page: 123
Last page: 125
License: This article is distributed under a Creative Commons Attribution License: https://creativecommons.org/licenses/by/4.0/
License URL: https://creativecommons.org/licenses/by/4.0/

==============================

## LT1. Bibliometric analysis: scale of Canadian librarian involvement in systematic review publications from U15 institutions

Janice Kung, Megan Kennedy
University of Alberta

**Introduction:** This is a follow-up study to a previous bibliometric analysis that analyzed the scale of librarian involvement in Systematic Reviews (SRs) at the University of Alberta. The intention of this project is to provide a Canada-wide analysis of health librarian involvement in SRs. There are several implications for this work; 1) training at a national level; 2) mentorship and coaching opportunities; 3) using librarian involvement as an indicator of quality SR publications, it could tell us whether or not Canadian SR publications are rigorously adhering to PRISMA-S and other reporting guidelines. **Methods:** Using Web of Science (WoS), we searched for SRs completed in the past five years. Systematic reviews identified through WoS will be screened in two phases: 1) Determining if the paper is a true SR publication (e.g. excluding duplicates, protocols, systematic review methodology papers, etc.), 2) Screening for librarian involvement (co-author, acknowledgement, or no involvement). **Results:** Of the 9514 studies retrieved, 7965 records advanced to full-text screening. A random sample of 400 references was pulled for data extraction. Of the 400 publications, 49 (12%) had a librarian co-author, 77 (19%) formally acknowledged librarians in the acknowledgements section, and 132 (33%) mentioned librarian support in the full-text of the paper. **Discussion:** This study will demonstrate the great deal of variation of how the work of librarians is reflected in SRs at a national scale. Continuing to educate researchers about the work of librarians is crucial to fully represent the value librarians bring to systematic reviews.

## LT2. Bringing order to chaos: how a work plan can help librarians support users doing systematic reviews and scoping reviews

Elizabeth Yates, Kymberly Ash
Brock University

Librarians who support faculty and students conducting evidence synthesis research can attest to the messiness of the process: these projects often stretch over long periods and there may be significant gaps in between consultation meetings, which make it difficult to stay on track. As well, the foundational steps in this type of research - developing an appropriate research question and designing a comprehensive search strategy - require a significant investment of time and expertise. Juggling multiple versions of separate documents adds to the confusion. To help support our users and enhance our efficiency, we developed a comprehensive work plan document which guides researchers through those key processes and forms a basis for meetings with researchers. This work plan is licensed for reuse and may be modified and adapted to suit other needs, for example, assisting graduate students with narrative literature reviews.

## LT3. From query to answer: building a health sciences FAQ space

Sabine Calleja, Eleni Philippopoulos

McGill University

**Objective:** Despite best efforts in teaching the basics of health sciences research, librarians receive a high volume of questions on the same topics from their students. To mitigate the repetition in answering these questions, which can take up valuable librarian and student time, a team of health sciences librarians at McGill University put together a health sciences FAQ hosted on the SpringShare LibApps software. **Methods:** Consisting of 22 initial questions organized among eight categories, including Knowledge Syntheses, Searching, Databases, Citations, Evidence-Based Practice and others, these FAQs transcend the specific health sciences disciplines. Questions were selected based on consultation with the team of health sciences librarians, and common themes were extracted and consolidated. Draft answers were compiled by the authors of the FAQ page and verified by all team members to ensure accuracy and compliance with library service guidelines. A centralized location for FAQs allows for more efficient searching and information retrieval, alleviating search fatigue. **Results:** The benefits of creating a health sciences FAQ allow our colleagues in other disciplines to find high-quality, vetted answers to common health sciences questions during their reference hours. Students are also encouraged to interact with the guide should they have additional questions, and their comments are taken into account when deciding on adding further questions. **Conclusions:** Though the FAQ was only launched in December 2023, we expect a high engagement rate with the guide.

## LT4. Try AI Day: helping librarians get acclimated to popular AI tools

Gary Atwood
University of Vermont

**Objectives:** Many health sciences librarians struggle to keep up with the rapid proliferation of artificial intelligence (AI) technology. Try AI Day provided University of Vermont librarians an opportunity to “test drive” a collection of several well-known AI tools. The primary goal was to discover the strengths and weaknesses of these tools through hands-on experimentation. **Methods:** Library faculty and staff were given a quick overview of AI’s growing role in academic life, prompting basics, and the workshop agenda. They then self-selected into groups. Each group was given credentials for a dummy Hotmail account and a Libguide URL (https://researchguides.uvm.edu/tryaiday) containing links to each tool along with some basic instructions. Groups had 60 minutes to explore. The facilitator was available to answer questions. The final 30 minutes were devoted to a debrief about each groups’ experiences and a brainstorming session about potential AI-related initiatives for the University Libraries. **Results:** Try AI Day was held on April 13, 2024, with 21 attendees. Survey results showed very positive ratings for both the format and content. Several suggested offering similar workshops to graduate students and research faculty. **Conclusions:** Try AI Day provided a positive, collaborative environment for librarians to begin experimenting with AI tools already being used by patrons. Hands-on exploration allowed attendees to discover tool capabilities and limitations firsthand.

## LT5. When lifecycles collide: embedding research data management into knowledge syntheses beyond the search

Angélique Roy, Meghan Goodchild, Courtney Svab
Queens University

**Objectives:** With the publication of PRISMA-S (Rethlefsen et al., 2021), there has been an increase in librarians' treatment of search strategies as data. In response to this, strategies are now being deposited into institutional, open access, and commercial repositories for increased transparency and reproducibility (Rod & Jill T. Boruff, 2024; Cunningham et al., 2023). Additionally, with the advent of open science initiatives and associated funding requirements such as the Tri-Agency Research Data Management (RDM) Policy (2021), researchers across all disciplines are increasingly recognizing the need to manage their data to support these goals. This research examines where and how search strategies as well as data from other stages of the systematic review lifecycle have been documented over the past five years as new trends for RDM have emerged. **Methods:** The authors performed a search for systematic reviews published in Canadian journals between 2019-2023 across three databases: Embase (Ovid) and MEDLINE (Ovid). A randomized, stratified sampling approach was used with a fixed number of articles per year (100 articles per year over 5 years). **Results:** Preliminary findings will be presented at the time of the conference as this research is currently in progress. **Discussion:** This research takes a birds-eye view of the application of RDM within the lifecycle of the systematic review to determine changing practices for sharing and preserving related data outputs. The authors aim to provide role-based recommendations to researchers and students undertaking knowledge syntheses. This research can help determine a practical approach for health sciences and RDM librarians to jointly guide researchers on developing a Data Management Plan by adopting and adapting the Data Management Plan Template: Systematic Reviews (Ganshorn et al., 2021).

JOURNAL INFORMATION
==============================
NLM Title Abbreviation: J Can Health Libr Assoc
Journal ID: J Can Health Libr Assoc
NLM Journal ID: 101229936
Journal ID: JCHLA

The Journal of the Canadian Health Libraries Association

EISSN: 1708-6892
Publisher: Canadian Health Libraries Association / Association des bibliotèques de la santé du Canada

ARTICLE INFORMATION
==============================
Subjects: LT = Lightning Talk

LT1. Bibliometric analysis: scale of Canadian librarian involvement in systematic review publications from U15 institutions

Kung Janice
Kennedy Megan
University of Alberta

JOURNAL INFORMATION
==============================
NLM Title Abbreviation: J Can Health Libr Assoc
Journal ID: J Can Health Libr Assoc
NLM Journal ID: 101229936
Journal ID: JCHLA

The Journal of the Canadian Health Libraries Association

EISSN: 1708-6892
Publisher: Canadian Health Libraries Association / Association des bibliotèques de la santé du Canada

ARTICLE INFORMATION
==============================
Subjects: LT = Lightning Talk

LT2. Bringing order to chaos: how a work plan can help librarians support users doing systematic reviews and scoping reviews

Yates Elizabeth
Ash Kymberly
Brock University

JOURNAL INFORMATION
==============================
NLM Title Abbreviation: J Can Health Libr Assoc
Journal ID: J Can Health Libr Assoc
NLM Journal ID: 101229936
Journal ID: JCHLA

The Journal of the Canadian Health Libraries Association

EISSN: 1708-6892
Publisher: Canadian Health Libraries Association / Association des bibliotèques de la santé du Canada

ARTICLE INFORMATION
==============================
Subjects: LT = Lightning Talk

LT3. From query to answer: building a health sciences FAQ space

Calleja Sabine
Philippopoulos Eleni
McGill University

JOURNAL INFORMATION
==============================
NLM Title Abbreviation: J Can Health Libr Assoc
Journal ID: J Can Health Libr Assoc
NLM Journal ID: 101229936
Journal ID: JCHLA

The Journal of the Canadian Health Libraries Association

EISSN: 1708-6892
Publisher: Canadian Health Libraries Association / Association des bibliotèques de la santé du Canada

ARTICLE INFORMATION
==============================
Subjects: LT = Lightning Talk

LT4. Try AI Day: helping librarians get acclimated to popular AI tools

Atwood Gary
University of Vermont

JOURNAL INFORMATION
==============================
NLM Title Abbreviation: J Can Health Libr Assoc
Journal ID: J Can Health Libr Assoc
NLM Journal ID: 101229936
Journal ID: JCHLA

The Journal of the Canadian Health Libraries Association

EISSN: 1708-6892
Publisher: Canadian Health Libraries Association / Association des bibliotèques de la santé du Canada

ARTICLE INFORMATION
==============================
Subjects: LT = Lightning Talk

LT5. When lifecycles collide: embedding research data management into knowledge syntheses beyond the search

Roy Angélique
Goodchild Meghan
Svab Courtney
Queens University

